# Quality of clinical management of children diagnosed with malaria: A cross-sectional assessment in 9 sub-Saharan African countries between 2007–2018

**DOI:** 10.1371/journal.pmed.1003254

**Published:** 2020-09-14

**Authors:** Jessica L. Cohen, Hannah H. Leslie, Indrani Saran, Günther Fink

**Affiliations:** 1 Harvard T. H. Chan School of Public Health, Boston, Massachusetts, United States of America; 2 Boston College School of Social Work, Chestnut Hill, Massachusetts, United States of America; 3 Swiss Tropical and Public Health Institute, Basel, Switzerland; 4 University of Basel, Basel, Switzerland; Liverpool School of Tropical Medicine, UNITED KINGDOM

## Abstract

**Background:**

Appropriate clinical management of malaria in children is critical for preventing progression to severe disease and for reducing the continued high burden of malaria mortality. This study aimed to assess the quality of care provided to children under 5 diagnosed with malaria across 9 sub-Saharan African countries.

**Methods and findings:**

We used data from the Service Provision Assessment (SPA) survey. SPAs are nationally representative facility surveys capturing quality of sick-child care, facility readiness, and provider and patient characteristics. The data set contained 24,756 direct clinical observations of outpatient sick-child visits across 9 countries, including Uganda (2007), Rwanda (2007), Namibia (2009), Kenya (2010), Malawi (2013), Senegal (2013–2017), Ethiopia (2014), Tanzania (2015), and Democratic Republic of the Congo (2018). We assessed the proportion of children with a malaria diagnosis who received a blood test diagnosis and an appropriate antimalarial. We used multilevel logistic regression to assess facility and provider and patient characteristics associated with these outcomes. Subgroup analyses with the 2013–2018 country surveys only were conducted for all outcomes. Children observed were on average 20.5 months old and were most commonly diagnosed with respiratory infection (47.7%), malaria (29.7%), and/or gastrointestinal infection (19.7%). Among the 7,340 children with a malaria diagnosis, 32.5% (95% CI: 30.3%–34.7%) received both a blood-test–based diagnosis and an appropriate antimalarial. The proportion of children with a blood test diagnosis and an appropriate antimalarial ranged from 3.4% to 57.1% across countries. In the more recent surveys (2013–2018), 40.7% (95% CI: 37.7%–43.6%) of children with a malaria diagnosis received both a blood test diagnosis and appropriate antimalarial. Roughly 20% of children diagnosed with malaria received no antimalarial at all, and nearly 10% received oral artemisinin monotherapy, which is not recommended because of concerns regarding parasite resistance. Receipt of a blood test diagnosis and appropriate antimalarial was positively correlated with being seen at a facility with diagnostic equipment in stock (adjusted OR 3.67; 95% CI: 2.72–4.95) and, in the 2013–2018 subsample, with being seen at a facility with Artemisinin Combination Therapies (ACTs) in stock (adjusted OR 1.60; 95% CI:1.04–2.46). However, even if all children diagnosed with malaria were seen by a trained provider at a facility with diagnostics and medicines in stock, only a predicted 37.2% (95% CI: 34.2%–40.1%) would have received a blood test and appropriate antimalarial (44.4% for the 2013–2018 subsample). Study limitations include the lack of confirmed malaria test results for most survey years, the inability to distinguish between a diagnosis of uncomplicated or severe malaria, the absence of other relevant indicators of quality of care including dosing and examinations, and that only 9 countries were studied.

**Conclusions:**

In this study, we found that a majority of children diagnosed with malaria across the 9 surveyed sub-Saharan African countries did not receive recommended care. Clinical management is positively correlated with the stocking of essential commodities and is somewhat improved in more recent years, but important quality gaps remain in the countries studied. Continued reductions in malaria mortality will require a bigger push toward quality improvements in clinical care.

## Abstract 2

Jessica Cohen and colleagues reveal the suboptimal malaria treatments that under 5's receive in sub-Saharan Africa.

## Author summary

**Why was this study done?:**

- Each year, millions of people become infected with malaria, and hundreds of thousands of people die from the disease, many of them young children in sub-Saharan Africa. Most deaths from malaria are avertable with timely provision of testing and treatment.
- Clinical guidelines for malaria include a blood test confirmation of disease prior to prompt treatment with effective antimalarials.
- Relatively little is known regarding provider compliance with these guidelines in malaria-endemic settings.

**What did the researchers do and find?:**

- We used nationally representative clinical observations of outpatient sick-child care from 9 sub-Saharan African countries to assess clinical management of children with a malaria diagnosis.
- We find that 32.5% of children diagnosed with malaria had both a blood test diagnosis and received appropriate antimalarial treatment, with a wide range of 3.4% to 57.1% across countries.
- Health worker training and facility supplies of diagnostic tests and effective antimalarials do not appear to explain these gaps in quality of care.
- Clinical management is somewhat better for 2013–2018 surveys than in the earlier years because of higher rates of blood test diagnosis.

**What do these findings mean?:**

- Despite increases in the distribution of malaria tests and effective antimalarial medications, significant gaps in the quality of care for pediatric malaria are present in these 9 countries.
- Further improvements in quality of malaria care may require a better understanding of remaining barriers and facilitators to appropriate management.

## Introduction

The year 2000 marked a turning point in global efforts to control malaria, which at the time was a leading cause of death among children under 5 in Africa [1]. Since then, malaria prevalence in sub-Saharan Africa has approximately halved; 19 countries globally have eliminated malaria altogether, and 20 more are on the verge of elimination [2,3]. This tremendous progress has been fueled by the widescale deployment of malaria prevention and treatment technologies that, together with unprecedented global malaria funding, have facilitated dramatic increases in the scale of malaria control efforts [3].

Despite this progress, the burden of malaria remains high, with over 200 million cases and an estimated 400,000 deaths per year [2]. Further, evidence suggests that progress has slowed in recent years, with some countries seeing increases in malaria transmission [2]. This has prompted the World Health Organization (WHO) and other global leaders in malaria control policy to call for a renewed focus on approaches to reducing the malaria burden. Further reductions in malaria morbidity and mortality will require new insights into gaps in the effectiveness of malaria control programs, including evidence on health system performance in the clinical management of pediatric malaria.

Most of the global malaria mortality burden is among young children in sub-Saharan Africa who are infected with the *Plasmodium falciparum* species of the parasite [2]. Children with *P*. *falciparum* malaria require prompt, appropriate antimalarial treatment in order to prevent progression of the disease to severe morbidity or death [4,5]. Since the symptoms of malaria overlap with a number of other common viral and bacterial diseases, blood test confirmation of the disease through microscopy or rapid test is strongly recommended [6,7]. WHO and national malaria control guidelines include blood test confirmation of malaria prior to treatment because testing can ensure better management of nonmalaria illness, reduce wasted resources on unnecessary antimalarial prescriptions, and improve health system surveillance data [6].

WHO and country-level guidelines for clinical management of malaria have evolved substantially in this century, including the introduction of Artemisinin Combination Therapies (ACTs) as first-line treatment for uncomplicated malaria between 2004–2006 and recommended testing prior to treatment for all ages between 2007–2012 [8]. Table 1 shows the recommended first-line treatment for uncomplicated and severe malaria for all countries in the sample, as well as the timing of guideline changes and the introduction of ACTs and rapid diagnostic tests (RDTs) [9–19]. Following these guideline changes, major efforts were made to improve access to testing and antimalarial treatment, including the free or heavily subsidized provision of ACTs to public sector health facilities and the large-scale distribution of malaria RDTs (Table 1). Evidence suggests that the scale-up of RDTs has contributed to higher rates of malaria testing [2,9,20] and that the widescale distribution of subsidized ACTs has contributed to declines in malaria morbidity and mortality [3,21], yet important gaps in clinical management of malaria remain [22–25].

**Table 1:** Malaria policy changes and introduction of ACTs and RDTs by country.

Survey		Treatment				Testing	
Country (survey year)	N sick children (N children with malaria diagnosis)	First-line treatment for severe malaria^1^	First-line treatment for uncomplicated malaria^1^	Year ACTs became first-line treatment for malaria^1^	Year ACTs became free/subsidized in public sector^1^	Year testing before treatment policy for all ages^1^	Year RDTs rolled out^2^
WHO^3^		AS	AL, AS + AQ, AS + MQ, AS + SP, DHA-PPQ	2006		2010	
Democratic Republic of the Congo (2018)	2,656 (2,027)	AS, QN	AS + AQ	2005	2006	2007	2011–2012
Ethiopia (2014)	1,898 (221)	AS, AM, QN	AL	2004	2004	2010	2010–2011
Kenya (2010)	1,999 (1,049)	AS, AM, QN	AL	2004	2006	2009	2012
Malawi (2013)	3,310 (954)	AS, QN	AL	2007	2007	2011	2011
Namibia (2009)	1,531 (110)	QN	AL	2006	2005	2012	2006
Rwanda (2007)	1,662 (713)	AS, QN	AL	2005	2016	2009	2011–2012
Senegal (2013–2017)	5,728 (102)	AS, QN	AL, AS + AQ, DHA-PPQ	2005	2010	2007	2007
Tanzania (2015)	4,950 (1,441)	AS, AM, QN	AL	2004	2006	2009	2009–2010
Uganda (2007)	1,022 (723)	AS, QN	AL	2004	2006	2008	2011–2012

^1^Source: World Malaria Report 2018, country profiles. https://www.who.int/malaria/publications/country-profiles/en/.

^2^Sources: [9–17].

^3^Sources: [18,19].

**Abbreviations**: AM, artemether; AL, artemether lumefantrine; AQ, amodiaquine; AS, artesunate; DHA-PPQ, dihydroartemisinin/piperaquine; MQ, mefloquine; NA, not applicable; QN, quinine; SP, sulfadoxine-pyrimethamine.

Several health system challenges are likely contributing to inadequate clinical management of the disease and thus to the enduring malaria mortality burden among young children [26]. First, caregivers often do not seek formal facility-based care for children with malaria symptoms, instead using over-the-counter medications, informal care, or no care at all [27–29]. Second, even children who are seen by a formal provider may not receive appropriate care [28]. Although empirical evidence on the role of provider quality in pediatric malaria deaths is limited [30–32], a recent study estimates that up to 50% of deaths from infectious diseases in low-income settings can be attributed to patients receiving low-quality care [33].

While a number of studies have assessed quality of care for malaria patients, most are based on interviews with patients or record reviews, though some studies at the national or subnational level also include direct observation of clinical practice [22,24,34–36]. One cross-country study that explores management of febrile illnesses using observation with a standardized checklist was conducted in the context of a program to improve clinical care with supportive supervision [37]. To our knowledge, no studies have used a uniform tool for clinical observation of malaria management across a range of countries to assess the quality of care for malaria under usual settings. This study uses clinical observation data from nearly 25,000 sick-child visits across 6,400 health facilities in 9 sub-Saharan African countries and spanning a 12-year period to evaluate the extent to which children with a malaria diagnosis received a blood test diagnosis and appropriate antimalarial treatment. We report estimates of these critical process measures of quality of care and explore potential predictors of quality, including facility stocking of essential malaria commodities and health worker training.

## Methods

### Data

The data used in this study are from the Service Provision Assessment (SPA) survey, implemented by ICF International [38]. All of the questionnaires can be found on the SPA website (https://dhsprogram.com/What-We-Do/Survey-Types/SPA-Questionnaires.cfm). We use data from 4 modules in the SPA survey battery: 1) direct observations of sick-child consultations for children under age 5, 2) facility surveys assessing service readiness, 3) interviews with healthcare providers, and 4) exit interviews with caregivers of the sick children observed in the consultation survey. All SPA surveys conducted in Africa in 2007 and later were analyzed, including the Democratic Republic of the Congo (2018), Ethiopia (2014), Kenya (2010), Malawi (2013), Namibia (2009), Rwanda (2007), Senegal (2013–2017), Tanzania (2015), and Uganda (2007). SPA surveys in Malawi and Namibia were a census of all registered public and private facilities in the country, whereas Rwanda was a full census of all public facilities and all large private facilities. In all other countries, the SPA survey was based on a nationally representative facility sample drawn from a master list and stratified by region and public/private ownership. All district-level and higher-level hospitals were included in the survey, and lower-level facilities were selected with a preset probability, with the lowest sampling fraction for the lowest tier facilities. In Senegal, health huts were administered a shorter assessment without observations of care and are therefore not included in the analysis. The SPA in Senegal was conducted annually for 5 years; we pool results across these years when presenting country-specific estimates. SPA surveys conducted before 2007 were excluded because they lacked sufficient detail on the type of antimalarial treatments prescribed.

The SPA surveys include direct observations of outpatient consultations with sick children under age 5. Sick children present on the assessment day were listed and sampled at a systematic interval after a random start number with the goal of sampling up to 5 observations per provider (maximum of 15 per facility). Direct observations were conducted by trained fieldworkers with clinical experience (typically nurses). Following the observation, providers reported to the interviewer their diagnosis and treatment plan. One of the diagnoses recorded in the SPA was a malaria diagnosis. Observers recorded whether the provider reported a malaria diagnosis (based on either RDT or microscopy) and also whether the provider reported that the malaria diagnosis was reached based on symptoms or based on a blood test. They also recorded which medications (if any) were prescribed or directly given to the child. SPA guidelines dictated that enumerators follow patients through their entire visit whenever possible [39]. An interview with the child’s caregiver was conducted after the observation for basic information on demographic characteristics, child symptoms, etc.

A provider interview and facility assessment were conducted on the same day as the clinical observation. We use information from the provider interview on the cadre and training in malaria diagnosis or treatment. From the facility assessment, we follow [40] and consider a facility to be equipped with malaria treatment and diagnosis equipment if the facility has observed, nonexpired artemether lumefantrine or artesunate-amodiaquine (the most common types of ACTs) in stock (any pack type) in the pharmacy area and had either observed, nonexpired RDTs in the laboratory or malaria service area and/or an observed microscope, blood slides, and staining equipment in the laboratory. These measures of observed, nonexpired equipment should be highly—but not perfectly—correlated with the availability of these commodities at the facility since it is possible that commodities were available in other locations (such as the child curative care area if separate from the malaria service area) or that providers are using expired medications or dosages.

A total of 25,426 sick-child observations were available in the SPA surveys. 274 observations were dropped because the child was 60 months or older. We also dropped 391 observations in which the SPA-supplied patient weight was missing or 0 (which indicates that either the clinical observation or caregiver interview were not consented to or not completed) and 5 observations in which the malaria diagnosis variable was missing.

### Analysis

The analysis was not based on a prespecified analysis plan. Observations were eligible for analysis if the provider reported diagnosing malaria, regardless of whether any other, additional, diagnoses were made (for example, respiratory infection). This inclusion criterion was used because SPA surveys only routinely inquire about malaria blood testing for those children ultimately diagnosed with malaria (not for children who had exclusively a nonmalaria diagnosis). WHO and country guidelines call for diagnostic confirmation of malaria by blood test and treatment with an ACT for uncomplicated malaria or parenteral artemisinin or quinine for severe malaria (Table 1) [6]. We assess the extent to which children with a malaria diagnosis receive 1) a blood-test–based diagnosis, 2) an appropriate antimalarial, and 3) both a blood test diagnosis and appropriate antimalarial.

The definition of these outcomes is restricted by several drawbacks in the SPA survey tool. First, for most SPA surveys, malaria test results are not reported—the SPA only includes a variable indicating whether or not a malaria diagnosis was based on a blood test. Specifically, for all children diagnosed with malaria, observers note whether the provider reports basing this decision on a blood test (either an RDT or microscopy) or based on symptoms only. While only available for the Tanzania (2015) and DRC (2018) surveys, observers in these surveys also checked malaria test results when a blood test diagnosis was reported and did confirm that 91.4% of these children had a record of a positive test. Second, the SPA surveys do not distinguish between diagnoses of severe or uncomplicated malaria. Accordingly, we considered a child to be treated with an “appropriate antimalarial” if they received an appropriate treatment for either uncomplicated or severe malaria, including oral ACTs, parenteral artemisinin, or injectable quinine. To the extent that children with uncomplicated malaria received treatment for severe malaria (or vice versa), this definition of “appropriate antimalarial” would be generous (an upper bound). The third limitation of the SPA surveys is that they do not report on the dosage of medication given, so we cannot determine whether the dose of antimalarial given was appropriate, again leading to the “appropriate antimalarial” definition being an upper bound of appropriate prescribing.

On the other hand, the SPA surveys do have detailed information about the types of antimalarials that were prescribed. We computed the fraction of children with a malaria diagnosis who received no antimalarial, an oral ACT, parenteral artemisinin, injectable quinine, oral artemisinin monotherapy, or another oral antimalarial (principally, chloroquine, amodiaquine, sulfadoxine-pyremethamine, and quinine). Oral artemisinin monotherapy is not recommended under any circumstances because of concerns about parasite resistance [41], and the “other antimalarials” are no longer recommended because of decreased efficacy [6]. We computed the fraction of children diagnosed with malaria who received each of these prescriptions as well as combinations of prescriptions for all combinations that included at least 10 cases. This excluded 24 malaria cases (0.25%).

We then assessed factors associated with receipt of both a blood test diagnosis and an appropriate antimalarial. We estimated a multilevel logistic regression model with a binary outcome variable equal to 1 if the child received both a blood test diagnosis and an appropriate antimalarial on a set of patient, caregiver, provider, and facility characteristics as well as survey year fixed effects. This analysis excludes an additional 385 observations (5%) because of missing covariates. We used cluster-robust standard errors to account for repeated measurements within facilities [42]. We use these adjusted models to predict receipt of both blood test diagnosis and appropriate antimalarial under the hypothetical scenario of all facilities having observed, valid ACTs and malaria testing equipment in stock, as well as all providers reporting training in malaria diagnosis and/or treatment.

Given the time it takes for countries to adapt to clinical guideline changes and the fact that ACTs and RDTs have become increasingly available in later years, the emphasis on testing and appropriate treatment (and access to tests and antimalarials) may be higher in recent years. Thus, for all outcomes, in addition to the pooled survey results, we report outcomes for the subset of country surveys conducted in 2013 and later (Malawi [2013], Senegal [2013–2017], Ethiopia [2014], Tanzania [2015], and Democratic Republic of the Congo [2018]). The 9 countries also have a range of health system structures and epidemiological profiles, so we perform a “leave one out” sensitivity analysis in S3 Table, in which we report results when dropping 1 country at a time from the data set.

All analyses were conducted using SPA-provided sampling weights, rescaled to maintain a weighted sample size equal to the observed sample size. The resulting estimates can be considered representative of all sick children under 5 seeking formal facility-based care in the countries included in our analysis at the time of the survey. All analyses were done using Stata Version 14 [43].

### Ethics statement

This analysis of secondary data was deemed exempt from human subject review by the Harvard Office of Human Research Administration (IRB15-3668), and no funding was received for this study. The study team did not seek informed consent from survey participants because the secondary data used in the study were anonymous, but the SPA surveyors obtain written consent from facility managers, providers, and patients when collecting the data.

## Results

A total of 24,756 sick-child clinical observations across 6,453 facilities were included in the pooled analysis (18,542 observations and 4,883 facilities within the 5-country subset from 2013–2018). Of all the facilities, 6.8% were hospitals and 70.6% were health centers (S1 Table). Most of the remaining facilities were dispensaries and lower-level clinics. 48.8% of facilities had at least one child with a malaria diagnosis in the sample. Among these facilities, 65.7% had observed and valid ACTs and malaria testing equipment in stock (79.7% in the 2013–2018 subsample) (S1 Table).

Sample characteristics for the pooled sample are presented in Table 2 and for the 2013–2018 subset in S2 Table. Overall, 7,340 children (29.6%) were diagnosed with malaria (25.6% in the 2013–2018 subset). Children diagnosed with malaria were, on average, 23.2 months old; the average age for the full sample was 20.5 months. Multiple diagnoses were common, with 42% and 15.6% of the children diagnosed with malaria also diagnosed with a respiratory infection or a gastrointestinal infection, respectively. Most malaria cases (85%) were treated outside of hospitals, in primary care health centers or smaller health posts, and only 7% were seen by a medical doctor or medical officer. 62.9% of malaria cases were seen by a provider with training in malaria diagnosis and/or treatment (62.6% in the 2013–2018 subset), and 67.9% were seen at a facility with observed malaria testing equipment and appropriate antimalarials in stock (80.3% in the 2013–2018 subset).

**Table 2:** Sample characteristics.

	Children with Malaria Diagnosis N = 7,340	Children without Malaria Diagnosis N = 17,416	Valid N
	N (%)	N (%)	
*Child characteristics*			
Age of child (months, mean ± SD)	23.2 ± 15.3	19.4 ± 15.4	24,382
Child is female	3,589 (49%)	8,248 (47.4%)	24,704
*Child diagnosis*			
Malaria	7,340 (100%)	0 (0%)	24,756
Respiratory infection	2,997 (42%)	8,031 (51.1%)	22,858
Gastrointestinal infection	1,111 (15.6%)	3,337 (21.3%)	22,858
*Caregiver characteristics*			
Caregiver age (years, mean ± SD)	28.3 ± 8.1	28.4 ± 8.4	23,581
Caregiver, primary education	3,764 (51.3%)	7,266 (41.7%)	24,756
Caregiver, some secondary education	1,896 (25.8%)	4,651 (26.7%)	24,756
*Facility ownership*			
Private facility	2,002 (27.3%)	3,283 (18.8%)	24,756
*Facility level*			
Hospital	1,101 (15%)	2,970 (17.1%)	24,756
Health center	4,390 (59.8%)	11,388 (65.4%)	24,756
Other (health post, dispensary, etc.)	1,849 (25.2%)	3,057 (17.6%)	24,756
*Facility stocking*			
Has observed/verified malaria testing equipment	5,514 (75.1%)	14,879(85.4%)	24,756
Has observed/verified appropriate antimalarial (ACT) in stock	6,524 (88.9%)	14,985 (86%)	24,756
Has both appropriate malaria testing equipment and antimalarial treatment	4,985 (67.9%)	13,137 (75.4%)	24,756
*Provider characteristics*			
MD or MO	509 (7%)	1,542 (8.9%)	24,476
Paramedical (for example, clinical officer, advanced practice clinician)	2,395 (33%)	6,869 (39.8%)	24,476
Nurse or other provider type (for example, CHW, aide)	4,364 (60%)	8,832 (51.2%)	24,476
Provider trained in malaria diagnosis or treatment	4,433 (62.9%)	10,785 (69.5%)	22,541

All estimates are weighted to be nationally representative of sick children under 5 seeking facility-based care in the year of the survey. **Abbreviations**: ACT, Artemisinin Combination Therapy; CHW, community health worker; MO, medical officer.

**Fig 1:**
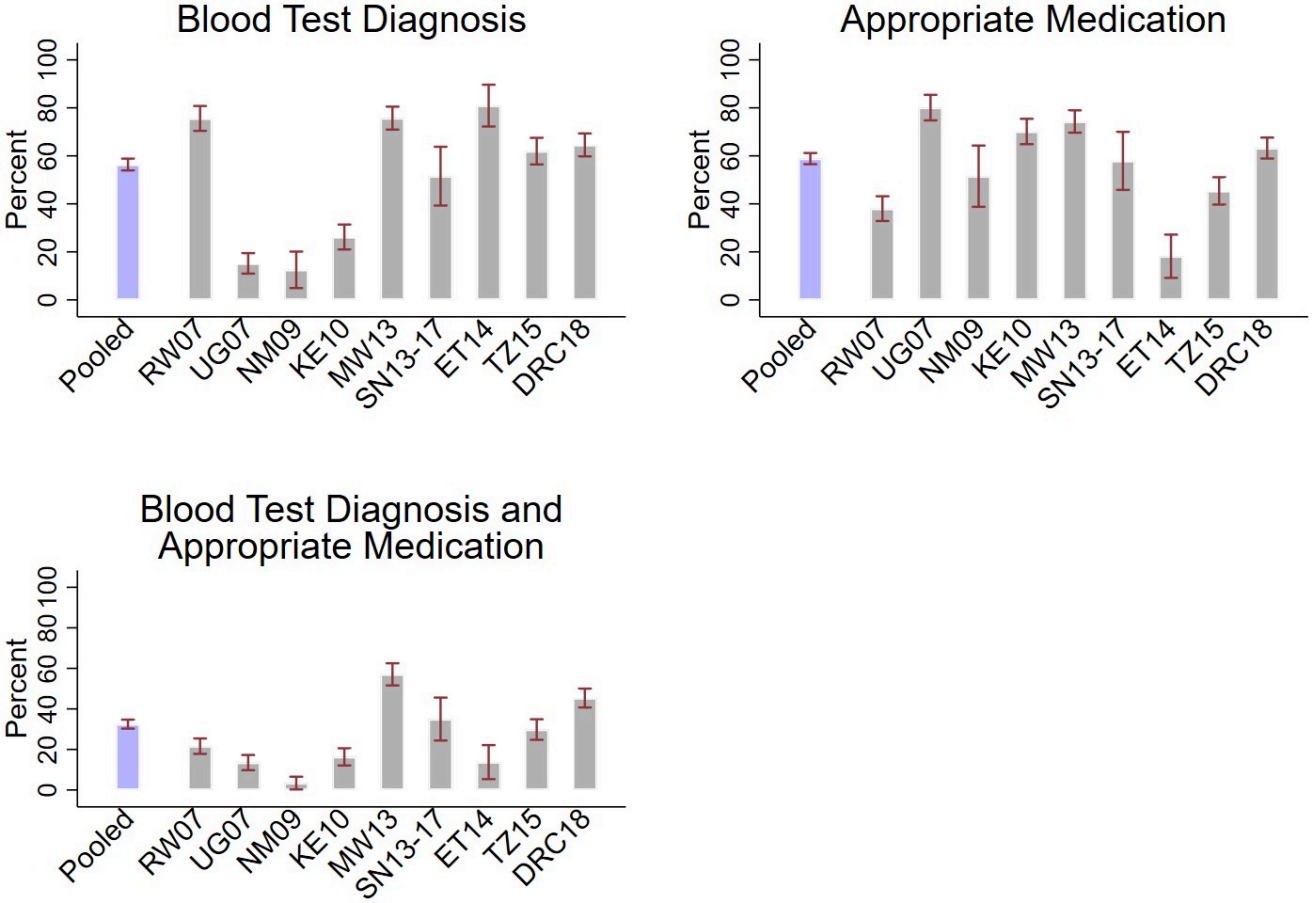
Clinical management of children diagnosed with malaria. Estimates are among the sample of children diagnosed with malaria. “Blood test diagnosis” indicates that the child’s malaria diagnosis was based on either a blood slide microscopy test or a rapid diagnostic test. “Appropriate medication” indicates that the child received either an oral ACT, parenteral artemisinin, or injectable quinine. For Senegal, all 5 survey rounds were combined. Data are weighted by SPA-supplied sampling weights to be nationally representative, and 95% confidence intervals are adjusted for clustering within facilities. ACT, Artemisinin Combination Therapy; DRC18, Democratic Republic of the Congo 2018; ET14, Ethiopia 2014; KE10, Kenya 2010; MW13, Malawi 2013; NM09, Namibia 2009; RW07, Rwanda 2007; SN13-17, Senegal 2013–2017; SPA, Service Provision Assessment; TZ15, Tanzania 2015; UG07, Uganda 2007.

Clinical management of children diagnosed with malaria is presented in Fig 1 and S3 Table. Among children diagnosed with malaria, 56.4% (95% CI: 53.9%–58.8%) received a blood test diagnosis overall, with a range of 12.5% (95% CI: 4.9%–20.1%) in Namibia to 80.9% (95% CI: 72.2%–89.6%) in Ethiopia. Among all children diagnosed with malaria, 58.8% (95% CI: 56.5%–61.2%) were prescribed an appropriate antimalarial, with prescribing ranging from 18.2% (95% CI: 9.2%–27.2%) in Ethiopia to 80.1% in Uganda (95% CI: 74.8%–85.4%). Overall, 32.5% (95% CI: 30.3%–34.7%) of children with a malaria diagnosis received both a blood test diagnosis and an appropriate antimalarial, ranging from 3.4% (95% CI: 0.3%–6.5%) in Namibia to 57.1% (95% CI: 51.6%–62.5%) in Malawi. When restricted to children seen at hospitals, 36.4% (95% CI: 31.1%–41.2%) received both a blood test diagnosis and appropriate antimalarial (S3 Table). These estimates are not very sensitive to dropping any particular country–year (S3 Table).

Blood test diagnoses appear to be more common in the later country surveys, although prescription of an appropriate antimalarial does not. Receipt of both a blood test diagnosis and appropriate antimalarial is somewhat higher in the 2013–2018 subset than in the pooled analysis, with 40.7% (95% CI: 37.7%–43.6%) of children diagnosed with malaria receiving both in these years (S3 Table).

**Fig 2:**
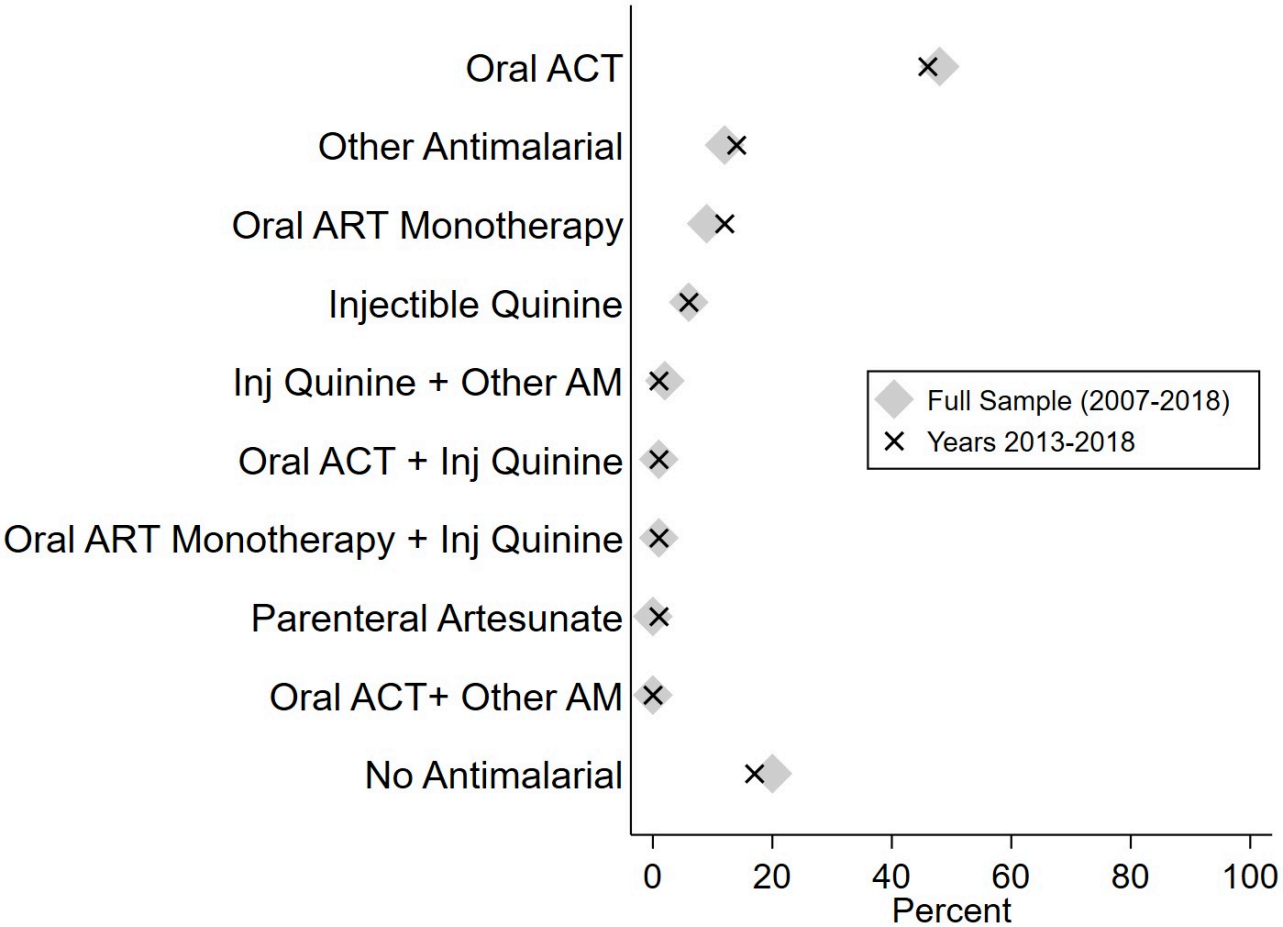
AM prescriptions among children diagnosed with malaria. “Other AM” includes chloroquine, sulfadoxine-pyremethamine, amodiaquine, quinine, and a few other very infrequently prescribed types of AMs not specified in the survey tool. Data are weighted using SPA-provided survey weights. ACT, Artemisinin Combination Therapy; AM, antimalarial; ART, artemisinin; SPA, Service Provision Assessment.

Turning to specific prescribing patterns, 20.1% (95% CI: 19.2%–21%) of the children diagnosed with malaria were not prescribed any antimalarial (Fig 2; country-specific results in S4 Table). 50.4% (95% CI: 49.3%–51.6%) were prescribed an ACT (48.1% prescribed an ACT alone and 2.3% in combination with other medications), and less than 0.5% were prescribed parenteral artemisinin. 9.7% (95% CI: 9.0% to 10.4%) of children with a malaria diagnosis were prescribed a quinine injection (6.0% alone and 3.7% in combination with other medications). Oral artemisinin monotherapy was prescribed to 9.7% (95% CI: 9.1%–10.4%) of children with a malaria diagnosis (9.2% alone and 0.5% in combination with other medications). Finally, 13.6% (95% CI: 12.8%–14.4%) of children with a malaria diagnosis were prescribed other, older antimalarials (11.8% alone and 1.8% in combination with other medications). Antimalarial prescribing patterns were very similar for the 2013–2018 subset as for the pooled analysis (Fig 2 and S4 Table), with 47.9% receiving an oral ACT, 16.9% receiving no antimalarial, and 16% receiving older antimalarials.

Results from multivariable logistic regressions are presented in Table 3 (crude ORs are in S5 Table). The odds of receiving a blood test diagnosis and an appropriate antimalarial were higher for older children than for infants. Children diagnosed with only malaria had 1.33 (95% CI: 1.09–1.61) higher odds of receiving a blood test diagnosis and an appropriate antimalarial than children diagnosed with both malaria and another illness. The odds of receiving a blood test diagnosis and an appropriate antimalarial were 3.67 (95% CI: 2.72–4.95) times higher for children being seen at facilities that had observed and valid malaria testing equipment in stock than for those who were not. This association was even stronger (OR 4.34; 95% CI: 2.66–7.10) for the 2013–2018 subsample. The association between being seen at a facility that had observed and valid ACTs in stock and receiving a blood test diagnosis and appropriate antimalarial was positive, but insignificant, in the full sample (1.31; 95% CI: 0.91–1.90) but somewhat stronger and significant in the 2013–2018 subsample (1.60; 95% CI: 1.04–2.46). No significant association was found between being seen by a provider with training in malaria diagnosis or treatment and receipt of a blood test and appropriate antimalarial. The odds of receiving both a blood test diagnosis and an appropriate antimalarial were significantly lower for children seen by a medical doctor or medical officer than children seen by a nurse but were higher for children seen by a paramedical provider (such as a clinical officer) than by a nurse in both the full sample and the 2013–2018 subsample.

**Table 3:** Patient, provider, and facility correlates with receipt of blood test diagnosis and recommended medication for malaria (logistic regression).

	All Years (N = 6,963)			2013–2018 Only (N = 4,496)		
	Adjusted OR	95% CI	p-value	Adjusted OR	95% CI	p-value
*Child characteristics*						
Age 12–23 months (ref: 0–11 months)	1.43	1.17–1.74	0.00	1.48	1.16–1.88	0.00
Age 24–35 months (ref: 0–11 months)	1.53	1.21–1.93	0.00	1.47	1.11–1.94	0.01
Age 36–47 months (ref: 0–11 months)	1.36	1.05–1.76	0.02	1.47	1.07–2.01	0.02
Age 48–60 months (ref: 0–11 months)	1.41	1.07–1.86	0.02	1.46	1.05–2.04	0.03
Female (ref: male)	0.98	0.84–1.13	0.76	1.02	0.85–1.22	0.83
*Child diagnosis*						
Malaria only (ref: malaria + other illness)	1.33	1.10–1.61	0.00	1.32	1.07–1.64	0.01
*Caregiver characteristics*						
Primary education (ref: no education)	0.94	0.77–1.15	0.54	0.97	0.76–1.23	0.78
Some secondary education (ref: no education)	0.78	0.60–1.02	0.07	0.71	0.53–0.96	0.02
*Facility ownership*						
Private facility (ref: public facility)	0.88	0.69–1.12	0.30	0.69	0.52–0.92	0.01
*Facility level*						
Hospital (ref: health post, dispensary)	1.1	0.75–1.61	0.62	1.10	0.70–1.74	0.67
Health center (ref: health post, dispensary)	1.15	0.81–1.63	0.45	1.18	0.79–1.77	0.42
*Facility stocking*						
Has valid/verified ACT in stock (ref: no valid/verified ACT in stock)	1.31	0.91–1.90	0.15	1.60	1.04–2.46	0.03
Has valid/verified malaria testing equipment (ref: no valid/verified malaria testing equipment)	3.67	2.72–4.95	0.00	4.34	2.66–7.10	0.00
*Provider characteristics*						
MD or MO (ref: nurse or other provider type)	0.47	0.33–0.68	0.00	0.52	0.35–0.76	0.00
Paramedical (for example, clinical officer, advanced practice clinician) (ref: nurse or other provider type)	1.45	1.04–2.03	0.03	1.61	1.05–2.46	0.03
Provider trained in malaria diagnosis or treatment (ref: provider never trained on malaria diagnosis or treatment)	0.85	0.69–1.06	0.15	0.85	0.66–1.09	0.21

Outcome variable is binary variable for diagnosis based on blood test and receipt of appropriate antimalarial. Coefficients are ORs from logistic regressions including all variables presented in table and survey year fixed effects. Standard errors are adjusted for clustering within facilities, and data are weighted using SPA-supplied sampling weights. **Abbreviations**: ACT, Artemisinin Combination Therapy; MO, medical officer; SPA, Service Provision Assessment.

**Fig 3:**
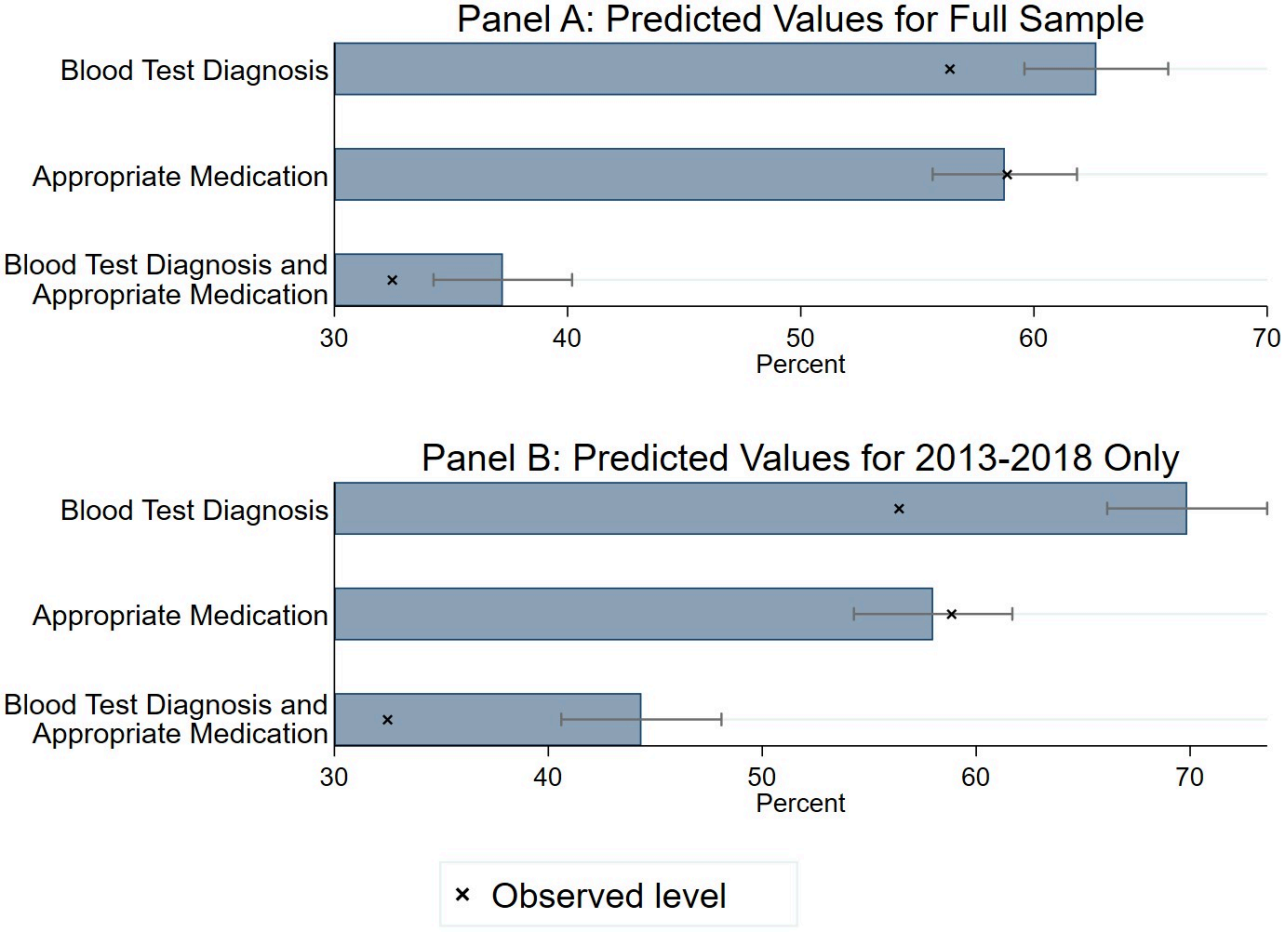
Estimated prevalence of clinical management of children diagnosed with malaria with universal stocking and provider training (N = 6,955 child visits). Estimates are among the sample of children diagnosed with malaria for which covariates included in the model presented in Table 3 are available. “Blood test diagnosis” indicates that the child’s malaria diagnosis was based on either a blood slide microscopy test or a rapid diagnostic test. “Appropriate medication” indicates that the child received an oral ACT, parenteral artemisinin, or injectable quinine. Predicted estimates are based on the model presented in Table 3, under the assumption that all children are seen at a facility that had observed, verified stocking of malaria diagnostic tests and ACTs and that all children are seen by a provider with training in malaria diagnosis and/or treatment, holding all other covariates at their mean value. Data are weighted by SPA-supplied sampling weights to be nationally representative, and 95% confidence intervals are adjusted for clustering within facilities. ACT, Artemisinin Combination Therapy; SPA, Service Provision Assessment.

Estimates from the logistic regressions presented in Table 3 imply that, if all children with a malaria diagnosis were seen at a facility that had observed ACTs and diagnostic testing equipment in stock and were seen by a provider with training in malaria management, a predicted 37.2% (95% CI: 34.3%–40.2%) of them would receive both a blood test diagnosis and an appropriate antimalarial in the pooled analysis (Fig 3). For the 2013–2018 subset, this predicted estimate increases modestly to 44.4% (95% CI: 40.6%–48.1%) of children with a malaria diagnosis receiving both a blood test diagnosis and appropriate medication. The difference between the observed and predicted levels is largest for receipt of a test and for receipt of both a test and appropriate medication, reflecting the strong association between test availability and testing shown in Table 3.

## Discussion

This paper uses a large clinical observation data set on malaria management to assess the quality of clinical care for children diagnosed with malaria in 9 sub-Saharan African countries. We find wide variation across countries in receipt of a blood test diagnosis and an appropriate antimalarial prescription. Overall, we find that two-thirds of children with a malaria diagnosis either did not receive a blood test diagnosis or were not given a recommended antimalarial—both essential process measures of quality of malaria care. While clinical management appears somewhat better in the 2013–2018 period—for diagnostic testing in particular—still, only about 40% of children with a malaria diagnosis are receiving both a blood test diagnosis and appropriate medication in these years, and 1 in 5 children with a malaria diagnosis do not get any antimalarial at all.

Diagnostic confirmation of malaria is a central recommendation of malaria treatment guidelines. Symptom-based diagnosis often yields high rates of antimalarial treatment for nonmalarial illnesses, which can lead to inappropriate ACT prescribing, wasted subsidy dollars, and increased likelihood of parasite resistance [44,45]. Nearly half of children with a malaria diagnosis in this study did not receive a diagnosis based on a blood test. The malaria diagnostic capacity of facilities in the countries surveyed was high, but not ideal, with over 20% of facilities lacking functioning diagnostic equipment. Being seen at a facility with observed diagnostic equipment was highly correlated with quality of care, suggesting that increased emphasis on testing and distribution of testing equipment is important for continued progress in malaria management. Diagnostic testing was also more common in the later survey years (2013–2018), which likely reflects the fact that RDTs were rolled out in the sample countries between 2010–2012 (Table 1) and that the emphasis on testing grew following the 2010 WHO guideline change to encourage parasitological confirmation of malaria prior to antimalarial treatment for all ages.

Treatment with ACTs is critical to preventing the progression of malaria to severe disease [4]; untreated severe malaria has an estimated case fatality rate of 13%–21% [5]. We find that roughly one-third of children diagnosed with malaria were either prescribed no antimalarial at all or were prescribed an antimalarial that is not recommended due to reduced efficacy such as chloroquine or sulfadoxine-pyrimethamine. Although parenteral artesunate is superior to injectable quinine for severe malaria [46], prescriptions for quinine injections were much more common than for parenteral artesunate. This likely reflects the limited availability of parenteral artesunate because in 2011, only 1 source had been prequalified by WHO [47]. Finally, we find that nearly 10% of children diagnosed with malaria were prescribed artemisinin monotherapy, which is strongly discouraged by WHO because of concerns about worsening parasite resistance [6,48]. Starting in 2007, WHO instructed member countries to begin phasing out monotherapy [41], so some prescribing of artemisinin monotherapy in the early survey years could be due to progressive phase-out. However, prescribing of artemisinin monotherapy persisted in the later surveys as well (S4 Table).

Stocking of ACTs was incomplete, with 85% of facilities having observed, valid ACTs in stock. Although ACTs became WHO’s recommended first-line treatment for malaria in 2006 and most of the study countries began making ACTs free or heavily subsidized in the public sector around the same time (Table 1), appropriate antimalarial prescribing was not different for the 2013–2018 subgroup than for the pooled estimates. Being seen at a facility with ACTs was positively associated with malaria management—although not significantly so in the full sample—suggesting that consistent stocking of ACTs is likely an enduring, important consideration in the quality of malaria management.

There are a number of reasons why healthcare workers may not be providing blood tests and appropriate antimalarials to children with a malaria diagnosis in the study countries. Testing may be incomplete because facilities lack a licensed microscopist or lab technician, because providers do not trust the tests or feel that their clinical judgment is superior, or because of high patient volumes and/or high out-of-pocket costs of the tests [34,35,49]. Health workers may not be prescribing appropriate antimalarials because the appropriate dosage or suspension is not available, because of provider or patient preference for alternative medications, or because of high out-of-pocket costs for these medications [49–51]. Some studies have found that patients continue to face out-of-pocket costs for health services and commodities that are intended to be provided free in settings similar to the study countries [52–54]. Continued improvements in the quality of care for malaria will require a deeper investigation into the lingering barriers and facilitators of appropriate clinical management of the disease.

While the availability of malaria tests and antimalarial medication was correlated with clinical management of malaria, provider training in malaria was not. While some previous research finds an association between health worker training and quality of care for malaria [55], training interventions have generally had small effects on provider malaria management [56–58] and on adherence to clinical guidelines more generally [59,60]. There are many factors beyond training that can influence provider adherence to guidelines, such as weak incentives and accountability, insufficient preservice education, high patient/provider ratios, or other health system factors [61–64]. Our study did not include a detailed measure of provider knowledge and competence, nor did it capture potentially important factors related to provider incentives, stress, and burnout. Estimates of these aspects of provider motivation alongside robust measures of quality of care are priorities for future research seeking to improve clinical management of malaria.

Our results are consistent with prior evidence from population-representative household surveys suggesting important gaps in diagnostic testing and ACT taking for children with malaria [22,25,65]. While these studies have the advantage of capturing children who are not brought for formal care, household surveys may be subject to recall bias and misunderstanding of clinical actions [66,67]. Our results underscore concerns about insufficient treatment for malaria and highlight the fact that even children who are brought to formal providers often receive inadequate care. Our results are also consistent with previous research on provider management of malaria, which has found that providers often fail to comply with testing and treatment guidelines but that compliance is somewhat responsive to guideline changes and the availability of equipment [9,14,68–75].

Our study was limited by the way in which the SPA survey records malaria testing, which is to indicate whether a child with a malaria diagnosis had a blood-test–based diagnosis or a clinical diagnosis. Because test results are not directly captured in SPA data, it is possible that the diagnosis may in some cases diverge from the test results. A number of previous studies have highlighted insufficient provider adherence to malaria test results [34,35,76]. While only available for the Tanzania (2015) and Democratic Republic of the Congo (2018) surveys, observers in these surveys checked malaria test results when tests were reported and did confirm that 91.4% of children diagnosed with malaria by blood test had a record of a positive test. Although it is possible that providers or lab technicians could have misrecorded test results in these settings, this is generally supportive that most children for whom the provider reported a blood-test–based diagnosis of malaria actually had a positive test result. The SPA also does not allow us to explore malaria testing for children who did not receive a malaria diagnosis (or whose malaria diagnosis was reported to be based on clinical symptoms), so we are unable to explore the important aspect of quality of care related to blood testing for all children with fever. Our assessment of quality of care for children diagnosed with malaria is, therefore, a narrow assessment of quality. Broader evaluations would include examinations and tests performed and their results, as well as provider counseling and communication.

Clinical observation data can be subject to Hawthorne effects and observer error [77,78]. For example, providers prescribing ACTs may have referred to them as “artemisinin,” resulting in misclassification of ACTs as oral artemisinin monotherapy. In the 2015 Tanzania SPA survey, observers confirmed 94.3% of observer-recorded ACT prescriptions and 93.7% of oral artemisinin monotherapy prescriptions using medical records. Another limitation of the SPA is that it reports on what medicines are prescribed, not necessarily which medicines are obtained or consumed. The SPA does, however, report whether the patient leaves the facility with medications in hand. 92% of children diagnosed with malaria are reported to leave the facility with at least one medication, although we cannot confirm that these are the same as those prescribed.

Patient flow through facilities could have influenced the quality of the observation data—for example, if enumerators were unable to follow patients from the provider exam to a lab for testing. SPA guidelines dictated that enumerators follow patients through their entire visit whenever possible [39]. Furthermore, in the surveys conducted after 2012, caregivers were asked in the exit interview whether they were sent to a lab within the facility or another provider for testing, and—among caregivers of patients who were recorded as having been diagnosed with malaria by blood test—78% of caregivers reported that they were sent for testing. This is likely a lower bound on the fraction of patients who actually were tested since it does not capture cases in which patients were tested by the primary provider within the exam room (for example, by RDT).

The countries in sub-Saharan Africa in which SPA conducted observations of care include more countries in East Africa than other parts of the subcontinent, and our results should be interpreted with this in mind. Furthermore, the countries included in the analysis have different epidemiological and health system profiles and may have been conducted in different seasons, which can have important implications for malaria prevalence and treatment. Although malaria epidemiology and management differ across the study countries, the overall results are not sensitive to the removal of specific surveys from the data set, suggesting that no particular country is driving the results. The results presented here are directly applicable to the 9 countries only. However, the consistent deficits in quality of care across these quite different countries suggests many other countries with high burden may experience similar challenges. High-quality evidence on the quality of malaria management—such as that based on observations of care—is urgently needed across a broader range of malaria-endemic countries, particularly those with the highest malaria burden that are the focus of the recent WHO “high burden to high impact” malaria strategy [79].

Two additional limitations to the study’s findings are the inability to draw firm conclusions regarding temporal changes in malaria management and the fact that regression estimates reflect correlations between patient-, provider-, and facility-level factors and clinical management, not necessarily causal effects. The estimates of predicted malaria management under the hypothetical scenario of all facilities having test equipment and antimalarials in stock are informative but could be biased by unobserved confounding factors related to both stocking and clinical management. The malaria landscape has evolved over the study period, but it is inappropriate to compare estimates of malaria management from earlier years to later years and make inference about changes over time since the surveys conducted in different years were also conducted in different countries. It is reasonable to infer from the comparison of the pooled estimates to those from 2013–2018 that testing has improved and that clinical management has improved modestly, but, again, any inference about changes over time is confounded by the changing sample composition. Given frequently changing guidelines, technological innovations, and investments in diseases such as malaria, integrating routine collection of healthcare quality data into health information systems is a key priority for quality monitoring and improvement [80].

### Conclusions

After many years of declining morbidity and mortality, malaria is at a crossroads [81]. Global funding for malaria programs has plateaued, and declines in malaria infection have stalled or possibly reversed. The results presented in this paper suggest that important gaps remain in the clinical management of malaria in the surveyed countries. Major efforts in quality of care will be needed to reduce the burden of malaria in the coming years.

## Supporting information

S1 Table Facility characteristics. (DOCX)

S2 Table Sample characteristics for 2013–2018 surveys only. (DOCX)

S3 Table Clinical management of children diagnosed with malaria: Estimates by time period, country, hospital, and leaving one country out at a time. (DOCX)

S4 Table Antimalarial prescriptions among children diagnosed with malaria. (DOCX)

S5 Table Patient, provider, and facility correlates with receipt of blood test diagnosis and recommended medication for malaria (logistic regression)—crude ORs. (DOCX)

## Abbreviations

ACT: Artemisinin Combination Therapy
RDT: rapid diagnostic test
SPA: Service Provision Assessment
WHO: World Health Organization
