# Supplementary material for: Quality of clinical management of children diagnosed with malaria: A cross-sectional assessment in 9 sub-Saharan African countries between 2007–2018
Source: PLoS Med. 2020 Sep 14;17(9):e1003254. doi: 10.1371/journal.pmed.1003254 (PMC7489507; doi:10.1371/journal.pmed.1003254)
Supplement: S1 Table — (DOCX) [file pmed.1003254.s001.docx]

| **Facility Characteristics** | | | | | | |
| --- | --- | --- | --- | --- | --- | --- |
|  | **Number of Facilities** | **Hospital** | **Health Center** | **Has Malaria Diagnostic Capacity** | **Has Artemisinin Combination Therapy** | **Has Malaria Diagnostic Capacity and Artemisinin Combination Therapy** |
|  | All Facilities | | | | | |
| All | 6,453 | 441 (6.8%) | 4557 (70.6%) | 5131 (79.5%) | 5514 (85.4%) | 4546 (70.5%) |
| Rwanda | 436 | 33 (7.6%) | 359 (82.3%) | 160 (36.7%) | 362 (83%) | 131 (30%) |
| Uganda | 319 | 19 (6%) | 300 (94%) | 96 (30%) | 248 (77.6%) | 77 (24.3%) |
| Namibia | 297 | 17 (5.7%) | 280 (94.3%) | 287 (96.6%) | 232 (78.1%) | 223 (75.1%) |
| Kenya | 518 | 48 (9.2%) | 158 (30.6%) | 243 (46.9%) | 463 (89.4%) | 222 (42.8%) |
| Malawi | 746 | 99 (13.2%) | 610 (81.7%) | 682 (91.5%) | 723 (97%) | 671 (90%) |
| Senegal | 1,602 | 55 (3.5%) | 1547 (96.5%) | 1517 (94.7%) | 1387 (86.6%) | 1360 (84.9%) |
| Ethiopia | 530 | 20 (3.8%) | 290 (54.7%) | 398 (75%) | 341 (64.3%) | 311 (58.7%) |
| Tanzania | 1,015 | 43 (4.3%) | 130 (12.8%) | 858 (84.5%) | 942 (92.8%) | 799 (78.8%) |
| DRC | 990 | 106 (10.7%) | 884 (89.3%) | 890 (89.9%) | 816 (82.4%) | 751 (75.9%) |
|  | Facilities with At Least One Malaria Diagnosis | | | | | |
| All | 3,151 | 269 (8.5%) | 2067 (65.6%) | 2305 (73.2%) | 2764 (87.7%) | 2071 (65.7%) |
| Rwanda | 274 | 16 (5.8%) | 232 (84.7%) | 105 (38.3%) | 240 (87.6%) | 90 (32.8%) |
| Uganda | 295 | 18 (6.1%) | 277 (93.9%) | 90 (30.5%) | 227 (77.1%) | 74 (25%) |
| Namibia | 57 | 3 (5.3%) | 54 (94.7%) | 53 (93%) | 56 (98.2%) | 52 (91.2%) |
| Kenya | 396 | 36 (9.1%) | 114 (28.8%) | 191 (48.3%) | 356 (89.8%) | 173 (43.7%) |
| Malawi | 481 | 59 (12.2%) | 391 (81.3%) | 435 (90.4%) | 468 (97.2%) | 428 (88.9%) |
| Senegal | 79 | 3 (4.3%) | 76 (95.7%) | 65 (81.8%) | 59 (74.4%) | 51 (64.9%) |
| Ethiopia | 115 | 4 (3.3%) | 65 (56.9%) | 104 (90.5%) | 87 (76.1%) | 85 (73.7%) |
| Tanzania | 565 | 28 (4.9%) | 72 (12.7%) | 465 (82.3%) | 542 (96%) | 449 (79.5%) |
| DRC | 889 | 103 (11.6%) | 786 (88.4%) | 797 (89.7%) | 729 (82%) | 670 (75.4%) |
