# Supplementary material for: Quality of clinical management of children diagnosed with malaria: A cross-sectional assessment in 9 sub-Saharan African countries between 2007–2018
Source: PLoS Med. 2020 Sep 14;17(9):e1003254. doi: 10.1371/journal.pmed.1003254 (PMC7489507; doi:10.1371/journal.pmed.1003254)
Supplement: S2 Table — (DOCX) [file pmed.1003254.s002.docx]

| **Sample Characteristics for 2013-2018 Surveys Only** | | | | | |
| --- | --- | --- | --- | --- | --- |
|  | **2013-2018 Only** | | | | |
|  | **Children with Malaria Diagnosis N=4,745** |  | **Children without Malaria Diagnosis N = 13,797** |  | **Valid N** |
|  | N (%) |  | N (%) |  |  |
| *Child Characteristics* |  |  |  |  |  |
| Age of child (months) | 23.7 ± 15.6 |  | 19.2 ± 15.4 |  | 18302 |
| Child is female | 2363 (48.8%) |  | 6616 (47.6%) |  | 18542 |
| *Child Diagnosis* |  |  |  |  |  |
| Malaria | 4745 (100%) |  | 0 (0%) |  | 18542 |
| Respiratory Infection | 1446 (31.1%) |  | 5635 (46.3%) |  | 16644 |
| Gastro-intestinal Infection | 604 (13%) |  | 2551 (20.9%) |  | 16644 |
| *Caregiver Characteristics* |  |  |  |  |  |
| Caregiver Age (years) | 28.5 ± 8.1 |  | 28.2 ± 8.1 |  | 17648 |
| Caregiver Primary Education | 2387 (49.3%) |  | 5746 (41.3%) |  | 18321 |
| Caregiver Some Secondary Education | 1352 (27.9%) |  | 3329 (24%) |  | 18321 |
| *Facility Ownership* |  |  |  |  |  |
| Private Facility | 1339 (27.6%) |  | 2507 (18%) |  | 18321 |
| *Facility Level* |  |  |  |  |  |
| Hospital | 775 (16%) |  | 2532 (18.2%) |  | 18321 |
| Health Center | 2804 (57.9%) |  | 8796 (63.2%) |  | 18321 |
| Other (health post, dispensary, etc.) | 1268 (26.2%) |  | 2570 (18.5%) |  | 18321 |
| *Facility Stocking* |  |  |  |  |  |
| Has Observed Malaria Testing Equipment | 4299 (88.7%) |  | 12563 (90.4%) |  | 18321 |
| Has Observed Appropriate Antimalarial (ACT) in Stock | 4328 (89.3%) |  | 12079 (86.9%) |  | 18321 |
| Has both Appropriate Malaria Testing Equipment and Antimalarial Treatment | 3891 (80.3%) |  | 11259 (81%) |  | 18321 |
| *Provider Characteristics* |  |  |  |  |  |
| MD or MO | 431 (8.9%) |  | 1364 (9.9%) |  | 18432 |
| Paramedical (e.g. Clin Officer, Adv Practice Clin) | 1863 (38.6%) |  | 6436 (46.7%) |  | 18432 |
| Nurse or other provider type (e.g. CHW, aide) | 2530 (52.4%) |  | 5988 (43.4%) |  | 18432 |
| Provider trained in malaria diagnosis or treatment | 2889 (62.6%) |  | 8783 (72.8%) |  | 16531 |
