# Supplementary material for: Quality of clinical management of children diagnosed with malaria: A cross-sectional assessment in 9 sub-Saharan African countries between 2007–2018
Source: PLoS Med. 2020 Sep 14;17(9):e1003254. doi: 10.1371/journal.pmed.1003254 (PMC7489507; doi:10.1371/journal.pmed.1003254)
Supplement: S3 Table — (DOCX) [file pmed.1003254.s003.docx]

| **Clinical Management of Children Diagnosed with Malaria: Estimates by Time Period, Country, Hospital and Leaving One Country Out at a Time** | | | | | | | |
| --- | --- | --- | --- | --- | --- | --- | --- |
|  | **Blood Test (%)** | **95% CI** | **Appropriate Medication (%)** | **95% CI** | **Blood Test & Appropriate Medication (%)** | **95% CI** | **N** |
| ***A. All*** | 56.4 | 53.9, 58.9 | 58.8 | 56.5, 61.2 | 32.5 | 30.3, 34.7 | 7340 |
| ***B. Post 2012 Only*** | 66.2 | 63.2, 69.1 | 57.2 | 54.2, 60.2 | 40.7 | 37.7, 43.6 | 4745 |
| ***C. Hospitals Only*** | 55.1 | 50.2, 60.1 | 60.2 | 55.4, 64.9 | 36.4 | 31.1, 41.6 | 2448 |
| ***D. By country:*** |  |  |  |  |  |  |  |
| *Rwanda* | 75.6 | 70.4, 80.8 | 38 | 32.9, 43.2 | 21.6 | 17.8, 25.4 | 713 |
| *Uganda* | 15.2 | 11, 19.5 | 80.1 | 74.8, 85.4 | 13.5 | 9.8, 17.3 | 723 |
| *Namibia* | 12.5 | 4.9, 20.1 | 51.5 | 38.8, 64.3 | 3.4 | 0.3, 6.5 | 110 |
| *Kenya* | 26.2 | 21, 31.4 | 70.2 | 64.9, 75.4 | 16.3 | 12.1, 20.6 | 1049 |
| *Malawi* | 75.7 | 70.9, 80.5 | 74.3 | 69.7, 79 | 57.1 | 51.6, 62.5 | 954 |
| *Senegal* | 51.5 | 39.3, 63.8 | 57.9 | 45.8, 70 | 35 | 24.4, 45.6 | 102 |
| *Ethiopia* | 80.9 | 72.2, 89.6 | 18.2 | 9.2, 27.2 | 13.7 | 5.4, 22.1 | 221 |
| *Tanzania* | 62 | 56.4, 67.5 | 45.4 | 39.7, 51.1 | 29.8 | 24.7, 34.9 | 1441 |
| *DRC* | 64.6 | 59.8, 69.3 | 63.3 | 58.9, 67.6 | 45.4 | 40.7, 50 | 2027 |
| ***D. Dropping One Country at a Time:*** | | | | | | | |
| *Without Rwanda* | 54.3 | 51.7, 56.9 | 61.1 | 58.6, 63.6 | 33.7 | 31.3, 36.1 | 6627 |
| *Without Uganda* | 59.7 | 57.1, 62.2 | 57.2 | 54.7, 59.6 | 34 | 31.6, 36.3 | 6617 |
| *Without Namibia* | 57.2 | 54.7, 59.6 | 59 | 56.6, 61.3 | 33 | 30.7, 35.2 | 7230 |
| *Without Kenya* | 61.8 | 59.2, 64.3 | 56.8 | 54.3, 59.4 | 35.4 | 32.9, 37.8 | 6291 |
| *Without Malawi* | 53.7 | 51, 56.4 | 56.7 | 54.2, 59.2 | 29 | 26.7, 31.3 | 6386 |
| *Without Senegal* | 56.5 | 54, 59 | 58.9 | 56.5, 61.2 | 32.4 | 30.2, 34.7 | 7238 |
| *Without Ethiopia* | 55.7 | 53.2, 58.2 | 60 | 57.7, 62.3 | 33 | 30.8, 35.3 | 7119 |
| *Without Tanzania* | 54.8 | 52.1, 57.5 | 62.8 | 60.4, 65.2 | 33.3 | 30.8, 35.7 | 5899 |
| *Without DRC* | 53.4 | 50.5, 56.2 | 57.2 | 54.5, 60 | 27.7 | 25.3, 30.1 | 5313 |
| Notes: Based on 24,756 direct observations across 6,453 facilities in 9 countries. All estimates are weighted to be nationally representative of sick children under 5 seeking facility based care in the year of the survey. | | | | | | | |
