# Supplementary material for: Quality of clinical management of children diagnosed with malaria: A cross-sectional assessment in 9 sub-Saharan African countries between 2007–2018
Source: PLoS Med. 2020 Sep 14;17(9):e1003254. doi: 10.1371/journal.pmed.1003254 (PMC7489507; doi:10.1371/journal.pmed.1003254)
Supplement: S5 Table — (DOCX) [file pmed.1003254.s005.docx]

| **Patient, Provider and Facility Correlates with Receipt of Blood Test Diagnosis and Recommended Medication for Malaria (Logistic Regression) - CRUDE ODDS RATIOS** | | | | | | | |
| --- | --- | --- | --- | --- | --- | --- | --- |
|  | **All Years  (N=6963)** | | |  | **2013-2018 Only  (N=4496)** | | |
|  | **Crude Odds Ratio** | **95% CI** | **p-value** |  | **Crude Odds Ratio** | **95% CI** | **p-value** |
| *Child Characteristics* |  |  |  |  |  |  |  |
| Age 12-23 months [ref: Age 0-11 months] | 1.41 | 1.168 - 1.708 | 0.00 |  | 1.45 | 1.148 - 1.818 | 0.00 |
| Age 24-35 months [ref: Age 0-11 months] | 1.49 | 1.188 - 1.866 | 0.00 |  | 1.41 | 1.076 - 1.841 | 0.01 |
| Age 36-47 months [ref: Age 0-11 months] | 1.30 | 1.013 - 1.677 | 0.04 |  | 1.41 | 1.042 - 1.909 | 0.03 |
| Age 48-60 months [ref: Age 0-11 months] | 1.40 | 1.068 - 1.834 | 0.01 |  | 1.43 | 1.041 - 1.966 | 0.03 |
| Female [ref: Male] | 0.98 | 0.850 - 1.128 | 0.77 |  | 1.02 | 0.862 - 1.212 | 0.80 |
| *Child Diagnosis* |  |  |  |  |  |  |  |
| Malaria Only [ref: Malaria+Other Illness] | 1.36 | 1.129 - 1.641 | 0.00 |  | 1.35 | 1.101 - 1.653 | 0.00 |
| *Caregiver Characteristics* |  |  |  |  |  |  |  |
| Primary Education [ref: No Education] | 0.98 | 0.805 - 1.181 | 0.80 |  | 0.98 | 0.774 - 1.228 | 0.83 |
| Some Secondary Education [ref: No Education] | 0.78 | 0.607 - 1.008 | 0.06 |  | 0.65 | 0.488 - 0.876 | 0.00 |
| *Facility Ownership* |  |  |  |  |  |  |  |
| Private Facility [ref: Public Facility] | 0.83 | 0.648 - 1.050 | 0.12 |  | 0.63 | 0.475 - 0.827 | 0.00 |
| *Facility Level* |  |  |  |  |  |  |  |
| Hospital [ref: Health Post, Dispensary] | 1.49 | 1.063 - 2.079 | 0.02 |  | 1.21 | 0.807 - 1.823 | 0.35 |
| Health Center [ref: Health Post, Dispensary] | 1.60 | 1.151 - 2.226 | 0.01 |  | 1.40 | 0.957 - 2.048 | 0.08 |
| *Facility Stocking* |  |  |  |  |  |  |  |
| Has Valid/Verified Artemisinin Combination Therapy in Stock [ref: No Valid/Verified ACT Medication in Stock] | 1.29 | 0.892 - 1.870 | 0.17 |  | 1.51 | 0.959 - 2.384 | 0.08 |
| Has Valid/Verified Malaria Testing Equipment [ref: No Valid/Verified Malaria Testing Equipment] | 3.63 | 2.724 - 4.825 | 0.00 |  | 3.96 | 2.468 - 6.354 | 0.00 |
| *Provider Characteristics* |  |  |  |  |  |  |  |
| MD or MO [ref: Nurse or Other Provider Type] | 0.44 | 0.318 - 0.621 | 0.00 |  | 0.44 | 0.304 - 0.624 | 0.00 |
| Paramedical (e.g. Clin Officer, Adv Practice Clin) [ref: Nurse or Other Provider Type] | 1.72 | 1.270 - 2.342 | 0.00 |  | 1.58 | 1.071 - 2.330 | 0.02 |
| Provider trained in malaria diagnosis or treatment [ref: Provider never Trained on Malaria Diagnosis or Treatment] | 0.87 | 0.708 - 1.079 | 0.21 |  | 0.88 | 0.691 - 1.132 | 0.33 |

Notes. Outcome variable is binary variable for diagnosis based on blood test and receipt of appropriate antimalarial. Coefficients are crude odds ratios from logistic regressions of the outcome on the binary variable indicated by row; if the independent variable is categorical (e.g. age), than each category is included in the regression. All regressions include survey-year fixed effects. Standard errors are adjusted for clustering within facilities and data are weighted using SPA-supplied sampling weights.
