# Supplementary material for: Quality of clinical management of children diagnosed with malaria: A cross-sectional assessment in 9 sub-Saharan African countries between 2007–2018
Source: PLoS Med. 2020 Sep 14;17(9):e1003254. doi: 10.1371/journal.pmed.1003254 (PMC7489507; doi:10.1371/journal.pmed.1003254)
Supplement: Supplementary file 5 [file pmed.1003254.s004.docx]

Antimalarial Prescriptions among Children Diagnosed with Malaria

	No Antimalarial	Oral ACT	Other Antimalarial (e.g. CQ, SP)	Oral Artemisinin Monotherapy	Injectable Quinine	Injectable Quinine + Other Antimalarial	Oral ACT + Injectable Quinine	Parenteral Artesunate	Oral ACT + Other Antimalarial	Oral ART Monotherapy + Injectable Quinine	N
A. All	20.1	48.1	11.8	9.2	6.0	1.3	1.9	0.4	0.5	0.5	7340
	(19.16 , 21)	46.92 , 49.2	(11.06 , 12.54)	(8.55 , 9.88)	(5.44 , 6.53)	(1.03 , 1.55)	(1.57 , 2.23)	(0.27 , 0.56)	(0.32 , 0.64)	(0.36 , 0.69)	
B. 2013-2018 Only	16.9	46.1	14.2	11.7	6.4	1.4	1.4	0.6	0.4	0.7	4745
	(15.86 , 18)	44.72 , 47.56	(13.23 , 15.21)	(10.74 , 12.56)	(5.71 , 7.11)	(1.09 , 1.77)	(1.05 , 1.72)	(0.4 , 0.85)	(0.19 , 0.53)	(0.44 , 0.9)	
C. By country:											
Rwanda (2007)	60.59	33.38	1.40	0.00	2.95	0.14	0.70	0.00	0.00	0.28	713
	(57 , 64.18)	29.91 , 36.85	(0.54 , 2.26)	(0 , 0)	(1.71 , 4.19)	(-0.13 , 0.41)	(0.09 , 1.31)	(0 , 0)	(0 , 0)	(-0.11 , 0.67)	
Uganda (2007)	4.09	64.15	15.47	0.35	8.88	1.74	1.90	0.00	3.20	0.00	723
	(2.65 , 5.53)	60.66 , 67.64	(12.82 , 18.12)	(-0.08 , 0.78)	(6.8 , 10.96)	(0.79 , 2.7)	(0.9 , 2.89)	(0 , 0)	(1.91 , 4.48)	(0 , 0)	
Namibia(2009)	12.23	49.53	8.10	27.13	0.00	1.00	0.00	0.00	0.00	0.00	110
	(6.08 , 18.38)	40.14 , 58.92	(2.99 , 13.22)	(18.78 , 35.48)	(0 , 0)	(-0.87 , 2.88)	(0 , 0)	(0 , 0)	(0 , 0)	(0 , 0)	
Kenya (2010)	16.14	58.02	6.59	6.88	5.36	1.23	5.13	0.00	0.00	0.39	1049
	(13.91 , 18.37	(55.04 , 61)	(5.09 , 8.09)	(5.35 , 8.41)	(4 , 6.72)	(0.56 , 1.89)	(3.79 , 6.46)	(0 , 0)	(-0.11 , 0.11)	(0.01 , 0.76)	
Malawi (2013)	4.06	60.44	3.10	18.48	6.71	1.03	3.44	0.72	0.00	1.85	954
	(2.81 , 5.31)	57.34 , 63.54	(2 , 4.2)	(16.01 , 20.95)	(5.12 , 8.3)	(0.39 , 1.67)	(2.28 , 4.6)	(0.18 , 1.25)	(0 , 0)	(0.99 , 2.71)	
Senegal (2013-2017)	19.68	44.00	18.18	4.24	7.84	1.83	0.00	1.39	1.19	0.00	102
	(11.92 , 27.44	34.32 , 53.68	(10.65 , 25.71)	(0.3 , 8.18)	(2.61 , 13.07)	(-0.77 , 4.44)	(0 , 0)	(-0.9 , 3.69)	(-0.93 , 3.31)	(0 , 0)	
Ethiopia (2014)	64.33	16.11	17.49	0.00	1.44	0.00	0.00	0.62	0.03	0.00	221
	(58 , 70.66)	11.25 , 20.97	(12.47 , 22.51)	(0 , 0)	(-0.14 , 3.02)	(0 , 0)	(0 , 0)	(-0.42 , 1.66)	(0.03 , 0.03)	(0 , 0)	
Tanzania (2015)	18.34	30.14	14.79	21.47	8.56	2.39	1.85	1.27	0.00	0.95	1441
	(16.34 , 20.34	27.77 , 32.51	(12.96 , 16.62)	(19.35 , 23.59)	(7.12 , 10)	(1.6 , 3.17)	(1.15 , 2.54)	(0.69 , 1.85)	(0 , 0)	(0.45 , 1.45)	
DRC (2018)	16.60	56.25	18.26	1.89	4.91	0.95	0.29	0.00	0.82	0.00	2027
	(14.98 , 18.22	54.09 , 58.41	(16.58 , 19.94)	(1.3 , 2.48)	(3.97 , 5.85)	(0.52 , 1.37)	(0.05 , 0.52)	(0 , 0)	(0.43 , 1.22)	(0 , 0)	

Notes: All estimates are weighted to be nationally representative of sick children under 5 seeking facility based care in the year of the survey.
